# Five Years of EVClub—From Journal Club to Worldwide Discussion Hub

**DOI:** 10.1002/jev2.70214

**Published:** 2025-12-14

**Authors:** Matilde Alique, Tom A. P. Driedonks, Ana Claudia Torrecilhas, Kenneth W. Witwer

**Affiliations:** ^1^ Departamento de Biología de Sistemas Universidad de Alcalá Madrid Spain; ^2^ Instituto Ramón y Cajal de Investigación Sanitaria (IRYCIS) Madrid Spain; ^3^ Department of CDL Research University Medical Center Utrecht Utrecht the Netherlands; ^4^ Instituto de Ciências Ambientais, Químicas e Farmacêuticas São Paulo Brazil; ^5^ Departamento de Ciências Farmacêuticas Laboratório de Imunologia Celular e Bioquímica de Fungos e Protozoários Universidade Federal de São Paulo (UNIFESP) São Paulo Brazil; ^6^ Department of Molecular and Comparative Pathobiology Johns Hopkins University School of Medicine Baltimore Maryland USA; ^7^ Department of Neurology Johns Hopkins University School of Medicine Baltimore Maryland USA

**Keywords:** ectosomes, exosomes, extracellular vesicles, journal club, microvesicles, science communication, social media

## 1 Introduction

A ‘journal club’ is a group meeting in which interested parties critically evaluate recent scientific literature in a specific field. Although the term was mentioned as early as the mid‐1800s (Topf et al. 2017), the first journal clubs as we know them were organized by Sir William Osler at McGill University in Montreal in 1875, encouraging collective reading of subscription journals to counter the prohibitively high cost of printed journals (Linzer 1987). In 1889, Osler was appointed Physician‐in‐Chief at the newly founded Johns Hopkins School of Medicine, where he laid the foundations for modern medical education, of which journal clubs were a part (National Library of Medicine n.d.). Today, journal clubs have been adopted by institutions around the world and often involve trainees presenting peer‐reviewed journal articles or preprints, followed by a discussion of methodology, findings and implications, guided by mentors. The process provides distinct educational benefits, including enhanced critical thinking skills, a deeper understanding of research methodology and greater awareness of research practices (Balamurali et al. 2024; McGlacken‐Byrne et al. 2020). Since the onset of the Coronavirus Disease 2019 (COVID‐19) pandemic, online or virtual journal clubs have increased in number, facilitated by advances in social media and digital technology. Unlimited by place, online journal clubs can maintain community interactions, enable knowledge sharing, and advance research globally.

## 2 Origins and Brief History of EVClub

The ‘Extracellular Vesicle Club’ (EVClub) grew out of a monthly microRNA and EV lunchtime journal club that Kenneth Witwer had founded with several other researchers at Johns Hopkins University in 2013. In March 2020, because of the COVID‐19 pandemic restrictions, Dr. Witwer converted the journal club into a weekly worldwide virtual event. Interested parties could (and still may) sign up for the mailing list (see https://www.surveymonkey.com/r/EVClubISEV) to receive login details. After moving online, EVClub grew into a global platform to discuss research and published articles, often including discussions with the authors themselves. In April 2021, EVClub was endorsed by the International Society for Extracellular Vesicles (ISEV). After a temporary hiatus beginning in February 2024, in October 2024, the EVClub made its return as a bi‐weekly gathering, with a ‘regular’ session every first Wednesday and a Special Interest Group session, either from Genitourinary System EVs (GUSEV) or Extracellular Vesicles in Nervous Systems (EViNS), every third Wednesday. Most meetings were recorded and can be rewatched anytime on ISEV's YouTube channel (www.youtube.com/@ExtracellularVesicleClub).

After 5 years of EVClub, it is time to assess the balance. What types of papers were featured, and from which journals? In which geographical chapter were the presenters based? How many people viewed the live and recorded sessions? And is EVClub still relevant? The following analysis covers the approximately 5‐year period from the founding of EVClub in March 2020 until June 2025, when this editorial was written.

## 3 EVClub by the Numbers

### 3.1 EVClub Subject Sources

Of 194 regular EVClubs as of June 2025, most involved one or more authors sharing their own peer‐reviewed publications, pre‐prints, or, more rarely, preliminary research findings. In 14 sessions, a presenter shared the work of another group. Approximately 190 papers have been presented on EVClub. Most EVClubs featured only one paper (171), while 12 included two or more papers. One session, in May 2023, featured seven groups studying exercise‐induced EVs. At least 15 EVClubs have consisted of topical lectures or discussions instead of focusing on specific publications or preprints.

### 3.2 Journals and Preprint Servers

EVClub has showcased research from around 70 journals or preprint servers. The most frequent source of papers is ISEV's Journal of Extracellular Vesicles (JEV), with nearly one‐third of the total papers (62). JEV is followed in volume by Nature Communications (7) and Cell (7), ISEV's Journal of Extracellular Biology (JExBio, 6), Proceedings of the National Academy of Sciences (PNAS) (4), Science Advances and Scientific Reports (4 each) and Advanced Science and Journal of Nanobiotechnology (3 each). Another 59 peer‐reviewed journals contributed one or two papers each. Of preprint servers, bioRxiv (an open‐access preprint repository for the biological sciences) was featured at least 17 times, and medRxiv (an online repository publishing preprints in all disciplines of the health sciences) twice. Most of the preprints on EVClub were later published in peer‐reviewed journals (not included in the numbers above). Results from four of five presentations of unpublished, unpreprinted work were also later published in journals.

### 3.3 Selection of Presenters and Topics

How were EVClub sessions chosen? And do they proportionately represent the depth and breadth of the EV field? As for most journal clubs, EVClub presenters and topics are chosen neither systematically nor randomly, and often with a great deal of subjectivity. The organizer(s) of any journal club have limited reading time and imperfect knowledge of a field and its literature, even when the field is less broad than that of EVs. Their choices are circumscribed by their own knowledge and biases, but also by time constraints. The first six EVClubs were presented by the organizer's collaborators or trainees simply because (1) he could reach them quickly and (2) they were willing to present on short notice. On other occasions, last‐minute replacements for cancellations were found in similar fashion. This approach introduces network bias but also allows an event to occur that would otherwise not. For 21% of EVClubs, the presenter(s) and topic(s) were chosen by a national society, special interest group, Student Network on EVs (SNEV), or another group. For the majority of sessions, the main organizer made the selection, drawing roughly equally on suggestions that were sent to him by community members, directly or through an online volunteer form (https://www.surveymonkey.com/r/EVPapers), and his personal review of the literature. Biases of one sort or another are likely; two strong biases bear mention. First, EVClub has featured articles in JEV and JExBio well out of proportion to the number of total EV articles that are published in ISEV's journals. Second, and although some occasional exceptions have been featured, chosen articles are typically felt to be consistent with the Minimal Information for Studies of Extracellular Vesicles (MISEV) guidelines (Théry et al. 2018; Welsh et al. 2024).

### 3.4 Division and Popularity of Topics

To understand the rough distribution of topics of EVClubs, we sorted presentations into one of five topics based on which we felt was most fitting: biomarkers, cell biology/basic science, EV mechanisms and roles in disease/physiology, EV therapeutics and methods/technology/guidelines. This exercise is fraught with subjectivity, as many sessions could be seen as fitting into more than one topic. Even so, the results suggest a strong emphasis on methodologies, techniques and guidelines in terms of percentage of sessions (#1), live participation (#2) and archive views (#3; see Table 1). One could argue that this emphasis is fitting for a general EV journal club, since techniques and methods may apply broadly across labs and subjects of inquiry. Parenthetically, almost 10% of EVClub sessions featured non‐mammalian EVs or the involvement of EVs in interactions between mammals and viruses or bacteria.

**TABLE 1:** Distribution of topics of EVClubs.

Main category	% of sessions	Live participation	Archive views
Biomarkers	9%	95	617
Cell biology/basic science	20%	111	729
Disease/physiology	22%	84	697
Methods, technology, guidelines	34%	105	775
Therapeutics	15%	81	610

### 3.5 Presenters/Discussants

**FIGURE 1:**
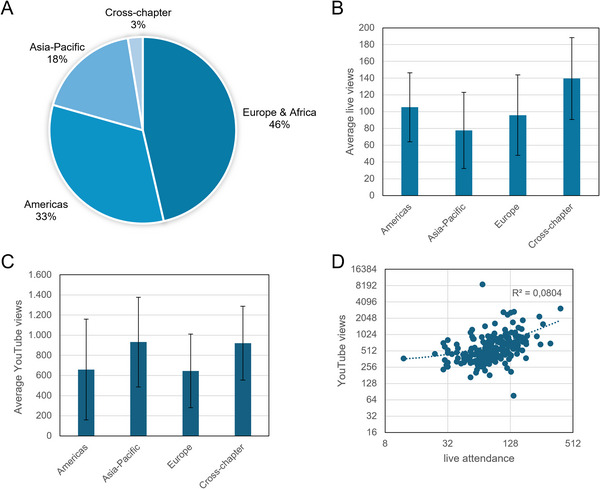
Statistics on EV club presenters and attendance. (A) EV club presenters by geographical ISEV chapter; (B) average number of live participants, by location of the EV Club presenter; (C) average number of YouTube views, by area of EV Club presenter; and (D) correlation between live attendance and YouTube views on a 2‐log scale (dotted line = best linear fit). Error bars (B, C) indicate standard deviation.

EVClubs feature a presenter, who also participates in a moderated discussion; in some cases, the presenter is joined after the presentation by additional discussants. The average EVClub session has had 1.6 presenters/discussants (range: 1–11). A total of 293 presenters/discussants have been involved as of June 2025. Two hundred and eighty‐one have contributed once, and 10 have contributed twice. The most frequently featured presenters/discussants were two of the founding leaders of ISEV's Rigor and Standardization Subcommittee (recently renamed to Reproducibility): Juan Manuel Falcón‐Perez (three times) and Rienk Nieuwland (four times). Of the 194 regular EVClubs, 46% had presenters from Europe/Africa, 33% from the Americas, and 18% from Asia‐Pacific. 3% of sessions included co‐presenters from multiple chapters (**Figure** **1A**). Gender balance of primary presenters has been almost exactly 50:50, and at least 122 EVClubs (>63%) had one or more presenters/discussants who were early‐career professionals. Breaking down the EVClubs by location of the presenters (**Figure** **1B**), cross‐chapter events attracted the largest average participation, at approximately 140, followed by the Americas (105), Europe/Africa (96) and Asia‐Pacific (78).

### 3.6 Moderators

The average EVClub session has had 1.3 moderators (range: 1–3), with around 30 people having served as moderators or co‐moderators. Of these, 14 were based in ISEV's Americas chapter, 9 in Europe/Africa, and 7 in Asia‐Pacific. Kenneth Witwer hosted and/or moderated all but four sessions. The next most frequent moderators were Minh Le and Wei Seong Toh, as part of a 10‐session collaboration with the Society for Clinical, Research and Translation of Extracellular Vesicles Singapore (SOCRATES). SNEV also figured prominently, contributing multiple co‐moderators across at least 16 collaborative sessions. Other national societies and local research networks or centres have also been featured on EVClub, including Grupo Español Innovación e Investigación en Vesículas Extracelulares (GEIVEX), Japanese Society for Extracellular Vesicles (JSEV), Italian Society for Extracellular Vesicles (EVIta), Austrian Society for Extracellular Vesicles (ASEV), Israeli Society for Extracellular Vesicles (IsREV), Slovenian network for extracellular vesicles (SiN‐EV), Latin America Extracellular Vesicles Network (LatinEV) the VES4US project (“Extracellular vesicles from a natural source for tailor‐made nanomaterials” from a European project funded by the Fet‐Open call of the Horizon 2020 Program) and the Vanderbilt Center for EV Research. Many of these collaborations have resulted in interviews and presentations with separate recordings on the EVClub YouTube channel.

### 3.7 Participation

Participation in EVClub was strong and consistent over the ∼5‐year period from March 2020 to June 2025. In total, there were nearly 19,000 logins to regular EVClub sessions, an average of almost 97 per event. Although the number of live session participants declined after the end of pandemic‐era restrictions (from an average per session of 139 and 115 in 2020 and 2021, respectively), participation has remained at healthy levels in 2022 (85), 2023 (67), 2024 (83) and 2025 (67), despite an 8‐month hiatus in 2024. The largest regular EVClub, with 389 participants, was a 2020 debate session on EV microRNAs, while the smallest session, in 2023, had only 12 participants. One hundred and sixty‐three EVClubs had more than 50 unique logins, and 80 had more than 100.

### 3.8 Time Slot Selection

One admitted drawback of EVClub is that live sessions inevitably exclude interested individuals in some parts of the world. The initial EVClubs were held at the same time as the predecessor, in‐person event (12 noon Eastern Time), and this time, once established, continued as the customary slot for around 75% of EVClubs. One could argue that consistency breeds habit and attendance; sessions held at the ‘usual’ time attracted an average of 103 participants, while EVClubs held at other times had significantly fewer participants (76, *p* < 0.0003). Pressure for times friendly for Asia‐Pacific was lower for the first 2 years of EVClub, since a weekly EV seminar forum, WebEVTalk, catered to the Asia‐Pacific audience during that period. Still, to avoid having all sessions at the same time, EVClub invitations included language such as, ‘Although the time is usually 12 noon Eastern Time, we are happy to adjust it to be more convenient for you’. The collaborations with SOCRATES and SNEV often held sessions at times more suitable for the Asia‐Pacific region. Almost 25% of EVClubs were thus held at times more convenient for the presenters and/or for regions that are usually underserved. Nevertheless, the EVClub founder apologises to those who have felt left out.

### 3.9 YouTube Archiving and Content

Considering that a perfect, consistent time for live events can never be achieved, EVClub uses YouTube to ensure that those who miss the events can watch them later. Recorded videos are edited and uploaded, usually within a week. All but two of the regular EVClub sessions are now publicly available. One recording failed for technical reasons, and another session was not posted because of unpublished data. Beyond the regular EVClub sessions, the EVClub platform has also been used for other events and communications. One was a 2021 virtual event co‐sponsored by ISEV and the Michael J. Fox Foundation for Parkinson's Disease, ‘The L1CAM controversy: Pulling down consensus’. Another recording featured a presentation by Edit Buzás at a 2023 meeting of the Chinese Society for Extracellular Vesicles (CSEV). Four ‘announcement’‐type recordings of EVClub or ISEV news and interviews with various EV scientists have been recorded. Finally, numerous Education Day videos, Urinary EV virtual symposium sessions, ISEV plenary sessions, an ‘EVClub Trailer’ video, the EV‐TRACK virtual workshop, and the 12 videos of ISEV's third massive online open course (MOOC) are available on the platform across multiple playlists.

### 3.10 Archive Viewership

The EVClub channel has greatly extended the reach of the live presentations. Altogether, the channel had received more than 200,000 views and almost 23,000 h of watch time as of June 2025, with more than 5000 subscribers. The scientific EVClub presentations, specifically (i.e., not including the ancillary materials), had >135,000 views, or approximately 710 per EVClub. Of presentations more than 1 year old, the minimum number of views was 245, and the maximum was 8606. According to YouTube statistics, the typical EVClub channel video garners between 190 and 300 views within 30 days of posting. Interestingly, videos from presenters in the ISEV Asia‐Pacific chapter have received the most views on average (932), just edging out presentations that feature scientists from multiple chapters (921) (**Figure** **1C**). Videos from presenters in the Americas and Europe/Africa have averages of 659 and 645, respectively. In 2024, most viewers were based in the United States (19.7%), followed by India (3.1%), Germany (0.9%) and Italy (0.7%). Most viewers were between 25 and 34 years old (79.2%) or slightly older (35–44 years, 10.6%). There is a weak correlation between live participation and number of viewers on YouTube (Pearson correlation = 0.28) (**Figure** **1D**), suggesting that papers that attract strong interest in live events sometimes, but not always, also attract archive viewership.

## 4 Member Reflections on EVClub

The following link contains comments and testimonials from ISEV Member Spotlights, which illustrate the value and community impact of EVClub. They highlight members’ experiences and perceptions.

https://www.isev.org/isev‐member‐spotlights‐isev‐tv

## 5 Concluding Remarks

Over 5 years since its inception, EVClub has evolved into a thriving global hub for exchanging ideas, discussing research and facilitating direct connections with authors and peers. Its consistent participation across ISEV chapters and its strong representation of early‐career researchers reflect a community that is deeply invested in open and inclusive science. What began as a small institution‐based initiative has evolved, through collaboration with ISEV, national societies and special interest groups, into a model for sustained virtual engagement in the EV field. In addition to featuring high‐impact papers from a wide range of journals, EVClub has played a key role in promoting ISEV's publications, including JEV and JExBio. Since JExBio published its first issue only in 2022, its representation in EVClub sessions has so far been limited, but this is expected to grow as the journal continues to expand its scope and visibility within the community.

As a platform that is free and open to all, EVClub complements the paid participation options of ISEV. ISEV has more than 2000 members, most with fee‐based membership, and hosts annual meetings that have attracted from 400 to 1800 participants. In contrast, EVClub offers regular events to a mailing list of 6000, with more than 5000 archive subscribers. Beyond the numbers, EVClub's true achievement lies in the sense of community it has fostered. The platform has demonstrated that scientific dialogue can flourish across time zones and disciplines when fuelled by curiosity, collegiality and a shared purpose. As new challenges and discoveries continue to shape the EV field, EVClub remains a living example of how collective effort and openness can advance knowledge and connection.

***Kenneth Witwer*** *: I would* *now like to use the first‐person voice to editorialize on my experience as the founder and main organizer. My first conclusion is that the EV community is the most vibrant, enthusiastic and committed scientific community I have been a part of. Despite the almost ridiculous variety of papers and topics we have featured, I can count on community members to show up. Some not only show up nearly every session but also ask insightful questions on all topics. Phil Askenase comes to mind immediately as perhaps the most devoted EVClub participant! But there are others*.

*Second, personal commitment from individuals is needed to keep EVClub going. I have had tremendous and appreciated help from others. To thank some of the most prolific helpers: Clotilde Théry, Metka Lenassi, Will Hotham, Camille Trinidad, Wei Seong Toh, Minh Le, Fabrice Lucien, Rienk Nieuwland, Juan Manuel Falcón, Stefano Pluchino and numerous current and former members of my lab, including co‐author Tom Driedonks, for hosting, co‐organizing and suggestions. However, EVClub is a labour of love, and a lot of labour, and sometimes it is one person who must roll up their sleeves and do it. From planning to maintaining the surveys and databases, sending the emails, to maintaining the YouTube channel, and of course hosting the sessions, it takes time. When I had to step back in February of 2024 for personal reasons, there was no single person or group of organizers who were ready or willing to take my place. Similarly, most collaborations with networks or societies run their course when individuals move on professionally or when decisions are made to start separate events. This is understandable, as everyone is overcommitted in science, but it emphasizes that essential time‐sensitive and repetitive tasks must often be tackled by one person rather than a committee or a far‐flung constellation of loose alliances*.

*And now a few questions. Will EVClub continue? Can I continue to run it indefinitely? Was it good to move to twice a month, or should we hold EVClub more or less frequently? Is it time for ISEV subgroups and non‐ISEV entities to start their own, separate EVClub‐like features? Certainly, several EV‐themed research seminars or journal clubs have been established in the last year or so by individual universities and societies. A selection of virtual events is healthy, but is it also still good to have an ISEV‐centralized event? I and my co‐authors encourage the ISEV leaders and community to ponder these questions and the value of continuing EVClub*.

## Author Contributions

**Matilde Alique**: project conception, writing – original draft, review and editing. **Tom A. P. Driedonks**: writing – original draft, review and editing, data collection and visualization. **Ana Claudia Torrecilhas**: writing – review and editing. **Kenneth W. Witwer**: writing – original draft, review and editing, data collection and visualization.

## Funding

M.A. was funded by Instituto de Salud Carlos III (ISCIII), co‐founded by Fondos Europeo de Desarrollo Regional (FEDER) (grant number PI19/00240) and by Universidad de Alcalá (“Ayuda de la Línea de Actuación Excelencia para el Profesorado Universitario de la UAH”; EPU‐INV‐UAH/2022/001). A.C.T. received support from Fundação de Amparo à Pesquisa do Estado de São Paulo, Brazil (FAPESP), grants 2019/15909‐0 and 2020/07870‐4, and Conselho Nacional de Desenvolvimento Científico e Tecnológico (CNPq) 408186/2018‐6. K.W.W. was supported by the Allen Frontiers Foundation, the Richman Family Precision Medicine Center of Excellence in Alzheimer's Disease at Johns Hopkins University, and the US National Institutes of Health under grants DA047807, CA241694, AI144997, and MH118164.

## Conflicts of Interest

K.W.W. is president of the International Society for Extracellular Vesicles and founder of EVClub; is or has been an advisory board member of B4 RNA, Everly Bio, Interactome Biotherapeutics, NeuroDex, and NovaDip; and holds stock options with NeuroDex. M.A., T.A.P.D., and A.C.T. declare no conflicts of interest.

## Data Availability

The data that support the findings of this study are available from the corresponding author upon reasonable request.
